# Successful treatment of locally advanced bulky cervical cancer complicated by irreducible complete uterine prolapse: A case report

**DOI:** 10.1097/MD.0000000000028664

**Published:** 2022-01-21

**Authors:** Dong Hyung Lee, Jong Kil Joo, Dong Soo Suh, Byung Sup Shin, Seo Yoon Hwang, Ki Hyung Kim

**Affiliations:** a Department of Obstetrics and Gynecology, Pusan National University School of Medicine, Busan, Republic of Korea; b Biomedical Research Institute, Pusan National University Hospital, Busan, Republic of Korea; c Department of Obstetrics and Gynecology, Hanmaeum Changwon Hospital, Changwon, Republic of Korea.

**Keywords:** advanced bulky carcinoma, cervical cancer, uterine prolapse

## Abstract

**Rationale:**

Cervical cancer complicated by irreducible complete uterine prolapse in elderly patients is extremely rare. No standard treatment has been established for these conditions.

**Patient concerns:**

A 74-year-old woman with a 30-year history of pelvic organ prolapse presented with irreducible complete uterine prolapse and a large exophytic mass involving the cervix and vaginal wall.

**Diagnosis:**

Biopsy of the mass was performed at the referring institution and showed invasive verrucous-type squamous cell carcinoma.

**Interventions:**

A prolapsed uterus with a tumor mass could not be manually reduced. After completion of concurrent chemoradiotherapy, the tumor mass in the prolapsed uterus decreased and could be reduced manually. Subsequently, the patient underwent hysterectomy and intra-abdominal uterosacral ligament suspension.

**Outcomes:**

At 19 months of postoperative follow-up, the patient remained disease-free and had no evidence of vault prolapse.

**Lessons:**

This study has important clinical implications and may provide a therapeutic strategy to address unmet medical needs in combination with locally advanced cervical cancer complicated by irreducible complete uterine prolapse. These conditions were successfully treated using a multidisciplinary approach of chemoradiotherapy followed by radical hysterectomy and uterosacral ligament suspension.

## 1 Introduction

Cervical cancer remains the most common gynecological malignancy worldwide, although its incidence and mortality rates have declined in most areas of the world during the past few decades. In Korea, it is the most common female genital malignancy; in 2017, the age-standardized incidence rate was 8.4 cases per 100,000 women. The numbers of newly diagnosed cervical cancer cases and deaths from cancer were 3469 and 868, respectively.^[1]^ Pelvic organ prolapse (POP) is a far more common disease than cervical cancer and is estimated to occur in 40% to 60% of parous women.^[2]^ Uterine prolapse is a common condition in elderly women and is mainly associated with increasing age, obesity, and high parity. Among parous women, 40% to 60% have varying degrees of POP and up to 20% require surgery during their lifetime.^[2,3]^

The concurrence of cervical cancer with irreducible uterine prolapse is rare. Treatment options for cervical cancer are well established according to disease stage. One of the challenges in the treatment of cervical cancer is the coexistence of uterine prolapse. Uniform management is not well established when these 2 conditions coincide.

Herein, we present an unusual case of locally advanced bulky squamous cell carcinoma of the cervix, complicated by irreducible complete uterine prolapse, in an elderly woman. The patient underwent chemoradiotherapy followed by modified radical hysterectomy and uterosacral ligament suspension. A multidisciplinary approach involving a gynecological oncologist, a urogynecologist, and a radiation oncologist resulted in successful outcomes.

## 2 Case presentation

**Figure 1:**
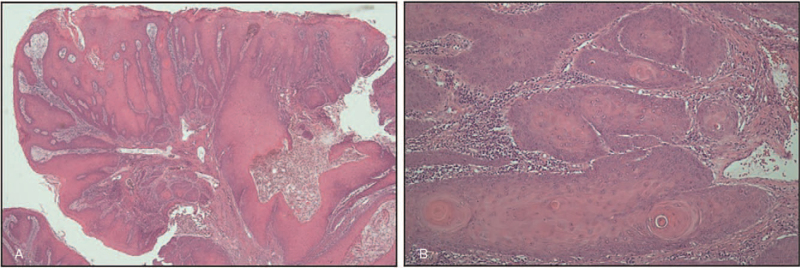
Microscopic findings of the squamous cell carcinoma (verrucous) of the cervix. (A) Exophytic acanthotic squamous epithelium shows pushing invasive growth (H&E, ×40); (B) tumor cells show abundant cytoplasm, minimal cytologic atypia, and rare mitoses (H&E, ×200).

**Figure 2:**
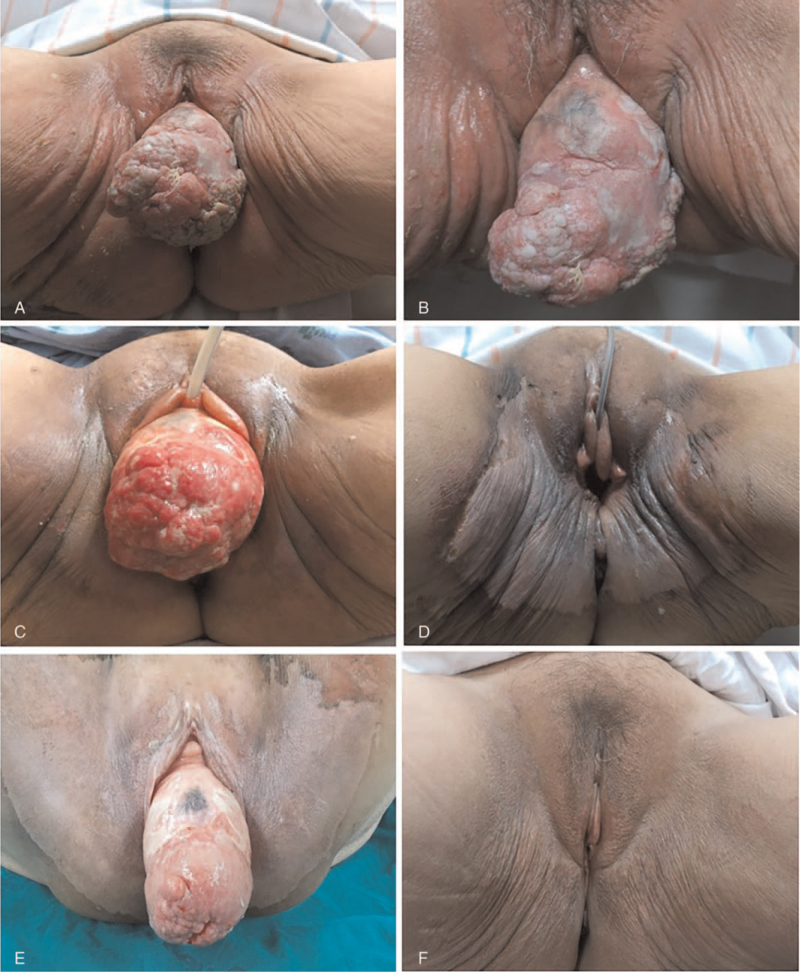
Pelvic examination findings. (A, B) Squamous cell carcinoma of the cervix and complete uterine prolapse at initial visit, (C) immediately after CCRT, (D) manually reduced state preoperatively, at 3 weeks after CCRT, (E) preoperatively before operation, at 4 weeks after CCRT, (F) postoperative, at 6 months. Radiation dermatitis of the genitalia was noted after the prolapsed uterus was irradiated (D&E). CCRT = concurrent chemoradiotherapy.

**Figure 3:**
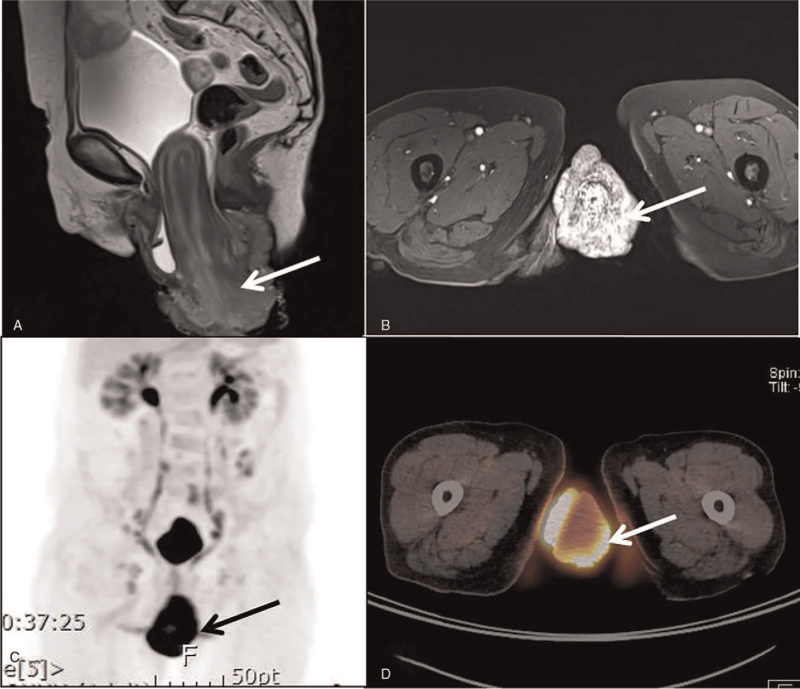
Imaging findings of squamous cell carcinoma (verrucous) of cervix and complete uterine prolapse. (A) T2-weighted sagittal magnetic resonance (MR) image showing complete prolapse of uterus and vagina (arrow). (B) Contrast-enhanced axial T1-weighted MR image showing marked enhancement in the prolapsed vagina and cervix (arrow). (C, D) FDG PET/CT images showing increased FDG uptake (arrow) in the prolapsed vagina and cervix. FDG = 18F-fluorodeoxyglucose, PET-CT = positron emission tomography-computed tomography.

A 74-year-old woman (gravida 5, para 4) with a known history of progressively worsening POP for approximately 30 years was referred to Pusan National University Hospital by another institution for uterine prolapse accompanied by an irregularly shaped large protruding mass. The patient reported an inability to reduce prolapse during the 3 months prior to the referral visit. The patient also complained of nocturia, associated with incomplete bladder emptying. Biopsy of the mass was performed by the referring institution and revealed an invasive verrucous squamous cell carcinoma (SCC) (Fig. 1). Pelvic examination revealed a large exophytic mass with complete uterovaginal prolapse (Fig. 2A and B). The prolapsed uterus with the tumor mass could not be reduced manually. She had a medical history of hyperlipidemia and surgical history of mastectomy for breast cancer. The patient's height and weight were 150 cm and 50 kg, respectively. The serum SCC antigen level at the initial visit increased (6.88 ng/mL). Neither high- nor low-risk human papilloma viruses (HPV) were detected at either the referring or our institution. Contrast-enhanced axial T1-weighted magnetic resonance imaging revealed an approximately 8.0 cm mass with marked enhancement in the prolapsed vagina and cervix (Fig. 3). Positron emission tomography-computed tomography (PET-CT) revealed avid 18F-fluorodeoxyglucose (FDG) uptake in the cervicovaginal region and the inguinal, external iliac, and obturator lymph nodes (Fig. 3). No evidence of distant metastases was observed. According to the International Federation of Gynecology and Obstetrics classification, the disease is clinically classified as stage IIIC1r.

Concurrent chemoradiotherapy (CCRT) consisting of 50.4 Gy whole pelvis irradiation in 28 fractions and 6 weeks of cisplatin (40 mg/m^2^/wk) was administered. On contrast-enhanced CT of the pelvis after CCRT completion, the tumor mass at the prolapsed uterus decreased (6.0 cm) and changed from a hard, irregular mass to a pinkish, soft smooth mass (Fig. 2C). The serum SCC antigen levels were normalized (1.09 ng/mL). At that time, the prolapsed uterus was manually reduced (Fig. 2D). Four weeks after CCRT, the mass had decreased significantly (4.5 cm) (Fig. 2E).

Subsequently, the patient underwent type II radical hysterectomy with bilateral salpingo-oophorectomy and intra-abdominal uterosacral ligament suspension without mesh placement. Pelvic lymphadenectomy was performed to assess the persistence of suspicious pelvic lymph nodes. No adhesions or fibrosis was observed in the operative field. Postoperative pathological examination showed residual SCC measuring 0.3 × 0.2 cm in size. The residual protruding masses in the uterine cervix and vagina were confirmed to be inflamed granulation tissues. Both the adnexa and pelvic lymph nodes were free of tumor cells. No lymphovascular invasion was observed. She tolerated radiation therapy and surgery well, and had no significant morbidity. At 19 months of postoperative follow-up, the patient remained disease-free and had no evidence of vault prolapse (Fig. 2F). Written informed consent was obtained from the participant for publication of the details of her medical case and any accompanying images.

## 3 Discussion

Cervical cancer complicated by complete uterine prolapse is rare. Squamous cell carcinoma of the female tract, the verrucous type (formerly named), is rare, described as a well-differentiated and slowly growing tumor, and is unlikely to metastasize to lymph nodes or distant areas.^[4]^ In 2020, the WHO classification of female genital tumors classified invasive squamous lesions into 2 main categories: HPV-associated and HPV-independent. The vast majority of cervical SCCs (>90–95%) are HPV-associated.^[5]^ Although the role of HPV in the etiology of this neoplasm has been suggested, HPV DNA has not detected in 75% of reported cases of SCC (verrucous type).^[4]^ A clear relationship between SCC, verrucous type, and HPV is yet to be described, and HPV infection tends to be infrequent in cases in which uterine prolapse is present.^[6]^ In our case, HPV was not detected at either the referring hospital or our institution. A systematic review has demonstrated the clinicopathological characteristics of these coexisting conditions.^[7]^ That review reported the mean age and median duration of prolapse were 63.7 years and 147.9 months, respectively, while the mean tumor size was 8.9 cm. Most patients were diagnosed with stage 1 disease (56.2%) and had an SCC type (83.9%). The pathophysiology of coexisting conditions remains unclear. Studies have shown that uterine prolapse itself may protect against cervical cancer or it can predispose to malignant transformation of the uterine cervix.^[8]^ Some authors have suggested that epithelial cornification during uterine prolapse may decrease the risk of malignant transformation.^[6,7]^ Others postulated that irritation of the cervix by being outside the body might promote the development of cancer. Women presenting with complete uterine prolapse are predisposed to a long process of chronic inflammation and direct mechanical irritation of the prolapsed areas, increasing the risk of cancer development.^[6,9]^ In this respect, the prolapse duration may be important for the development of cancer. Prolapse for >10 years may be associated with an increased risk of cancer development.^[10]^ In our case, the patient had progressively worsening POP that persisted for approximately 30 years. The patient did not seek medical attention because of uterine prolapse; thus, cervical carcinoma was not diagnosed until the disease had progressed. Owing to its rarity, no standard recommendations have been established for the treatment of these coexisting conditions. Optimal management options include surgery, radiotherapy (RT) alone, or a combination of these 2 modalities. Treatment strategies vary considerably among reports and should be tailored according to the stage of cervical cancer and degree of pelvic prolapse. Surgery should be considered as the preferred option whenever possible to avoid serious visceral injuries from RT. In addition, surgical treatment for POP can effectively improve health-related quality of life. Matsuo et al^[8]^ reported treatment patterns and survival outcomes in patients with cervical cancer complicated by complete uterine prolapse. Surgery alone or RT alone was performed in 33.3% and 38.5% of patients, respectively, while neoadjuvant RT followed by surgery was performed in 11.5% of patients. Survival outcomes were more favorable with surgery-based treatment than with radiation-based treatment, in terms of recurrence-free survival and disease-specific overall survival. Surgery alone or in combination with RT as a neoadjuvant or adjuvant method was considered the preferred option in 72% of cases of cervical cancer with irreducible uterine prolapse.^[7]^ In cases where immediate surgery is not feasible, chemoradiotherapy followed by surgery with curative intent has been reported to have good results in terms of both oncological and functional outcomes,^[11,12]^ as in our case. Some authors have mentioned that when external beam radiotherapy (EBRT) is planned, reduction of uterine prolapse and hysterectomy are recommended before EBRT to reduce the risk of visceral injury. Karateke et al^[13]^ managed vaginal cancer and a massive uterovaginal prolapse that could not be repositioned under general anesthesia, but was repositioned by surgical intervention prior to RT. Despite this recommendation, Reimer et al^[11]^ reported successful EBRT treatment for an irreducible uterovaginal prolapse combined with cervical cancer, a case similar to ours. In our case, after the completion of chemoradiotherapy, the prolapsed uterus became reducible, and a minimal residual tumor was confirmed after surgery. We performed CCRT followed by salvage hysterectomy and uterosacral ligament suspension to control locally advanced cervical cancer (LACC) and irreducible complete uterine prolapse. CCRT is the standard treatment for LACC. However, the 5-year-overall survival (OS) remains at approximately 70%, and histopathologic residual tumors are observed in 40% to 50% of patients. Salvage hysterectomy is a treatment specifically for patients in whom tumors persist after definitive RT/CCRT. Gosset et al^[14]^ reported that salvage hysterectomy causes a high rate of postoperative complications (approximately 25%) and this surgical procedure did not prevent locoregional or distant recurrences. Conversely, Takekuma et al^[15]^ demonstrated that salvage hysterectomy for patients with persistent cervical cancer after definitive RT/CCRT reduced the mortality rate by approximately 60% compared with systemic chemotherapy and could be a curative treatment for these patients. PET-CT may play a role in the selection of patients for salvage hysterectomy following CCRT for LACC.^[16,17]^ Further prospective clinical trials on salvage HT after RT or CCRT are required. The benefits of adjuvant hysterectomy (AH) after definitive CCRT for LACC are controversial because of unclear survival benefits and associated complications (Table 1).^[18–23]^ Three recent meta-analyses compared survival outcomes between CCRT followed by AH and CCRT alone.^[21–23]^ An updated review by Lu et al^[21]^ demonstrated that AH after CCRT may positively affect survival outcomes. In contrast, 2 previous meta-analyses reported no difference in the OS between the 2 groups.^[22,23]^ Most gynecologic oncologists agree that AH may be effective in patients with persistent residual disease after CCRT. The major clinical issue associated with surgical treatment after RT/CCRT is the high incidence of postoperative complications in the irradiated pelvis, which must be balanced against the potential benefits of treatment. Complication rates depend on the radicality of the surgery, accompanying procedures, tumor stage, residual tumor, time interval from CCRT completion to AH, and the surgeon's skill.^[22]^ Routine use of AH should be avoided because of its significant morbidity.

**Table 1:** Studies comparing survival outcomes between CCRT followed by hysterectomy and CCRT alone.

Author	Study design	Subjects	Comparison	Outcome in CCRT + HT group
Albert et al (2019)	Retrospective	IB2 to IIA2 cervical cancer	CCRT + HT vs CCRT alone	No difference in OS
Yang et al (2020)	Retrospective	Locally advanced cervical adenocarcinoma	CCRT + HT vs CCRT alone	Improve OS and PFS
Takekuma et al (2020)	Retrospective	Persistent cancer after RT/CCRT	RT/CCRT + HT vs RT/CCRT + CT	Improve OS and PFS
Yoshida et al (2020)	Retrospective	IB2 to IIB cervical cancer	CCRT + HT vs CCRT alone	Improve OS and PFS reduce the risk of recurrence
Shim et al (2018)	Meta-analysis	Locally advanced cervical cancer	CCRT + HT vs CCRT alone	Not improve OS, reduce the risk of recurrence
Shi et al (2018)	Meta-analysis	Locally advanced cervical cancer	RT/CCRT + HT vs RT/CCRT + CT	Not improve OS, reduce the risk of recurrence
Lu et al (2021)	Meta-analysis	Locally advanced cervical cancer	CCRT + HT vs CCRT alone	Improve OS and PFS (subgroup analyses^∗^ did not show significant benefit) reduce the risk of recurrence

In conclusion, our study has important clinical implications and may provide a therapeutic strategy to address unmet medical needs in combined conditions, such as LACC complicated by irreducible complete uterine prolapse. To date, no standard treatment for these conditions has been established. This case presents an unusual combination of these 2 conditions, leading to a treatment dilemma. In our case, chemoradiotherapy followed by salvage hysterectomy was effective, without adverse events. This approach may be a treatment option for locally advanced bulky cervical carcinoma and irreducible complete uterine prolapse in carefully selected patients. Physicians should be aware of the optimal treatment for uterine prolapse with cervical cancer, because these coexisting conditions may become more prevalent.

## 4 Patient consent statement

The patient has provided informed consent for the publication of this case.

## Author contributions

**Conceptualization:** Dong Hyung Lee, Ki Hyung Kim.

**Resources:** Byung Sup Shin.

**Supervision:** Jong Kil Joo.

**Writing – original draft:** Dong Hyung Lee.

**Writing – review & editing:** Dong Soo Suh, Seo Yoon Hwang, Ki Hyung Kim.

## References

[1] Hong S Won YJ Park YR Jung KW Kong HJ Lee ES. Cancer statistics in Korea: incidence, mortality, survival, and prevalence in 2017. Cancer Res Treat 2020;52:335–50. 32178489 10.4143/crt.2020.206 PMC7176962

[2] Smith FJ Holman CD Moorin RE Tsokos N. Lifetime risk of undergoing surgery for pelvic organ prolapse. Obstet Gynecol 2010;116:1096–100. 20966694 10.1097/AOG.0b013e3181f73729

[3] Maher C Feiner B Baessler K Schmid C. Surgical management of pelvic organ prolapse in women. Cochrane Database Syst Rev 2013;4:CD004014. 10.1002/14651858.CD004014.pub5 23633316

[4] Dane B Dane C Erginbas M Baran S Cetin A. Verrucous carcinoma of the cervix in a case with uterine prolapse. Ann Diagn Pathol 2009;13:344–6. 19751912 10.1016/j.anndiagpath.2009.02.005

[5] International Agency for Research on Cancer, WHO Classification of Tumours Editorial Board. Female Genital Tumours. 5th ed 2020.

[6] Bacalbasa N Halmaciu I Cretoiu D. Radical hysterectomy for cervical cancer in patients with uterine prolapse. In Vivo 2020;34:2073–8. 32606185 10.21873/invivo.12010 PMC7439874

[7] Matsuo K Fullerton ME Moeini A. Treatment patterns and survival outcomes in patients with cervical cancer complicated by complete uterine prolapse: a systematic review of literature. Int Urogynecol J 2016;27:29–38. 25971615 10.1007/s00192-015-2731-8 PMC7528440

[8] Loizzi V Cormio G Selvaggi L Carriero C Putignano G. Locally advanced cervical cancer associated with complete uterine prolapse. Eur J Cancer Care (Engl) 2010;19:548–50. 19694800 10.1111/j.1365-2354.2008.01010.x

[9] Du XL Tao J Sheng XG. Intensity-modulated radiation therapy for advanced cervical cancer: a comparison of dosimetric and clinical outcomes with conventional radiotherapy. Gynecol Oncol 2012;125:151–7. 22198339 10.1016/j.ygyno.2011.12.432

[10] Pardal C Correia C Serrano P. Carcinoma of the cervix complicating a genital prolapse. BMJ Case Rep 2015;24:2015. 10.1136/bcr-2015-209580 PMC4458593 26009601

[11] Reimer D Sztankay A Steppan I. Cervical cancer associated with genital prolapse – a brief review of the literature and long-term results of successful treatment with radiochemotherapy and surgery in a very frail patient. Eur J Gynaecol Oncol 2008;29:272–5. 18592794

[12] Ishibashi N Maebayashi T Asai-Sato M Kawana K Okada M. Radiation therapy for vaginal cancer in complete uterine prolapse with intrauterine adhesion: a case report. BMC Womens Health 2019;19:69. 31122220 10.1186/s12905-019-0767-5 PMC6533701

[13] Karateke A Tugrul S Yakut Y Gürbüz A Cam C. Management of a case of primary vaginal cancer with irreducible massive uterine prolapse--a case report. Eur J Gynaecol Oncol 2006;27:528–30. 17139994

[14] Gosset M Chargari C Bentivegna E. Should we cease to perform salvage hysterectomy after chemoradiation and brachytherapy in locally advanced cervical cancer? Anticancer Res 2019;39:2919–26. 31177130 10.21873/anticanres.13421

[15] Takekuma M Takahashi F Mabuchi S. Propensity score-matched analysis of systemic chemotherapy versus salvage hysterectomy for persistent cervical cancer after definitive radiotherapy/concurrent chemoradiotherapy. BMC Cancer 2020;20:1169. 33256667 10.1186/s12885-020-07672-w PMC7708164

[16] Rajasooriyar C Lin MY Kalra R Lim A Narayan K. The role of positron emission tomography in the selection of patients for salvage hysterectomy following chemoradiotherapy for locally advanced cervical cancer. Int J Gynecol Cancer 2019;29:266–71. 30630887 10.1136/ijgc-2018-000088

[17] Phippen NT Havrilesky LJ Barnett JC Hamilton CA Stany MP Lowery WJ. Does routine posttreatment PET/CT add value to the care of women with locally advanced cervical cancer? Int J Gynecol Cancer 2016;26:944–50. 27051057 10.1097/IGC.0000000000000705

[18] Allbright R Lee A Vijayakumar S. Preoperative chemoradiation followed by hysterectomy for cervical cancer: patterns of care and survival in a large, hospital database. J Gynecol Oncol 2019;30:e41. 30887759 10.3802/jgo.2019.30.e41 PMC6424845

[19] Yoshida K Kajiyama H Yoshihara M. The role of additional hysterectomy after concurrent chemoradiation for patients with locally advanced cervical cancer. Int J Clin Oncol 2020;25:384–90. 31552530 10.1007/s10147-019-01551-6

[20] Yang J Yang J Cao D Shen K Ma J Zhang F. Completion hysterectomy after chemoradiotherapy for locally advanced adeno-type cervical carcinoma: updated survival outcomes and experience in post radiation surgery. J Gynecol Oncol 2020;31:e16. 31912674 10.3802/jgo.2020.31.e16 PMC7044008

[21] Lu W Lu C Yu Z Gao L. Chemoradiotherapy alone vs. chemoradiotherapy and hysterectomy for locally advanced cervical cancer: a systematic review and updated meta-analysis. Oncol Lett 2021;21:160. 33552278 10.3892/ol.2020.12421 PMC7798101

[22] Shim SH Kim SN Chae SH Kim JE Lee SJ. Impact of adjuvant hysterectomy on prognosis in patients with locally advanced cervical cancer treated with concurrent chemoradiotherapy: a meta-analysis. J Gynecol Oncol 2018;29:e25. 29400018 10.3802/jgo.2018.29.e25 PMC5823986

[23] Shi D Liang Z Zhang C Zhang H Liu X. The effect of surgery on the survival status of patients with locally advanced cervical cancer after radiotherapy/chemoradiotherapy: a meta-analysis. BMC Cancer 2018;18:308. 29558900 10.1186/s12885-018-4232-x PMC5859532

